# Disseminated herpes zoster with pulmonary lesions mimicking miliary tuberculosis in an HIV-infected patient previously treated with adalimumab: A case report

**DOI:** 10.1016/j.idcr.2026.e02732

**Published:** 2026-08-18

**Authors:** Shuhei Tada, Kazuaki Fukushima, Yota Aizawa, Shota Kodaira, Seowoong Jung, Masaru Tanaka, Taiichiro Kobayashi, Ryoko Sekiya, Atsushi Ajisawa, Akifumi Imamura

**Affiliations:** a Department of Infectious Diseases, Tokyo Metropolitan Cancer and Infectious Diseases Center, Komagome Hospital, 3-18-22 Honkomagome, Bunkyo-ku, Tokyo, 113-8677, Japan; b Department of Infection Prevention and Control, Tokyo Metropolitan Cancer and Infectious Diseases Center, Komagome Hospital, 3-18-22 Honkomagome, Bunkyo-ku, Tokyo, 113-8677, Japan; c Survey and Analysis Section, Infectious Disease Control Division, Bureau of Public Health, Tokyo Metropolitan Government, 2-8-1 Nishishinjuku, Shinjuku-ku, Tokyo, 163-8001, Japan

**Keywords:** Disseminated herpes zoster, Miliary tuberculosis, Bilateral pulmonary nodules, Tumor necrosis factor-alpha inhibitor, Human immunodeficiency virus, Acquired immunodeficiency syndrome

## Abstract

Disseminated herpes zoster (DHZ) in immunocompromised patients is uncommon but potentially life-threatening. Pulmonary involvement is particularly rare and can mimic miliary tuberculosis, complicating the diagnosis. We report herein a 51-year-old, male, Japanese patient with a human immunodeficiency virus (HIV) infection, uncontrolled diabetes mellitus, and hidradenitis suppurativa in whom DHZ developed five months after he discontinued adalimumab therapy. He presented with a five-day history of fever, abdominal pain, and generalized vesicular eruptions. Chest computed tomography revealed small, diffusely distributed, bilateral nodules suggestive of miliary tuberculosis. However, acid-fast bacillus tests repeatedly returned negative. Endoscopic evaluation revealed multiple, gastric ulcerations, and immunohistochemical staining of gastric ulcer biopsy specimens confirmed a varicella-zoster virus infection. Intravenous acyclovir rapidly improved the cutaneous lesions, and the pulmonary nodules resolved almost entirely within 18 days. The gastrointestinal ulcers also healed with the antiviral therapy. The present case highlights the importance of considering DHZ in the differential diagnosis of diffuse, pulmonary nodules in immunocompromised patients, especially those with an HIV infection and a history of biologic therapy. Prompt diagnosis, appropriate, radiological follow-up, and early administration of antiviral therapy are crucial to ensuring a favorable clinical outcome.

## Introduction

Disseminated herpes zoster (DHZ), defined by the presence of more than 20 vesicular lesions outside the primary and adjacent dermatomes, is often associated with viremia caused by the varicella-zoster virus (VZV) [1]. Although DHZ may involve visceral organs, such as the liver, pancreas, and central nervous system [2], pulmonary involvement is rare and can radiologically resemble miliary tuberculosis, making diagnosis challenging [3].

Tumor necrosis factor-alpha (TNF-α) inhibitors, including adalimumab, are well-established risk factors of both herpes zoster and tuberculosis [4], [5]. While the risk is significantly high in the first year of treatment [6], the duration of heightened susceptibility following treatment discontinuation remains unclear.

Herein we report a patient with a human immunodeficiency virus (HIV) infection in whom DHZ with pulmonary lesions developed five months after he discontinued adalimumab therapy. This case highlights the importance of considering DHZ in the differential diagnosis of diffuse, pulmonary nodules in immunocompromised patients, particularly those with an HIV infection and a history of biologic therapy.

## Case presentation

A 51-year-old, male, Japanese patient was referred to the study center with severe epigastric pain and generalized, vesicular eruptions. His past medical history included type 2 diabetes mellitus, hypertension, hidradenitis suppurativa, oral candidiasis, and secondary syphilis. He had received a diagnosis of hidradenitis suppurativa ten years previously,for which a one-year course of adalimumab was prescribed because his condition was refractory to conventional therapy. However, the treatment was discontinued five months prior to the current presentation following the development of drug-induced pneumonia, and treatment with bimekizumab, a monoclonal IgG1 antibody targeting interleukin−17A and interleukin-17F, was begun two weeks before admission. He then developed progressive vesicular eruptions; notably, severe epigastric pain had begun four days before the onset of the characteristic skin eruptions and had persisted for five days.

On admission, his height and weight was 170.7 cm and 80.6 kg, respectively. He had lost 10 kg of body weight during the preceding months. His temperature was 37.7°C, pulse 120 beats/min, oxygen saturation 97% on ambient air, and blood pressure 165/95 mmHg. He was alert and fully oriented. A physical examination revealed erythematous papules and vesicles with a red halo distributed over the trunk and extremities, raising suspicion of DHZ. Immunochromatography (DermaQuick® VZV) of a sample of blister fluid was positive for the VZV antigen. White plaques were observed on the posterior pharyngeal wall and tongue indicating oral candidiasis. The bilateral axillary and inguinal regions demonstrated confluent nodules with purulent discharge and epithelialized skin tunnels indicating hidradenitis suppurativa. Abdominal tenderness was present in the epigastric region, and no neurological abnormalities were observed. Table 1 summarizes the laboratory test results. Tests for HIV demonstrated CD4 cell count 165/μL and HIV-RNA 8.4 × 10^4^ copies/mL, indicating advanced immunosuppression. HIV infection was newly diagnosed during the current hospitalization. Multiplex polymerase chain reaction with a FilmArray® Meningitis/Encephalitis Panel confirmed the positivity for VZV.

**Table 1:** Laboratory test results at hospital admission.

Laboratory test	Values
**Blood**	
Leukocyte count	6000 /μL
CD4 count	165 /μL
Hemoglobin	10.2 g/dL
Platelet count	8.7 × 10^4^ /μL
Creatinine	0.89 mg/dL
BUN	8 mg/dL
Sodium	140 mEq/L
Potassium	3.4 mEq/L
AST	43 U/L
ALT	48 U/L
Total bilirubin	0.8 mg/dL
CRP	8.89 mg/dL
HIV-RNA	8.4 × 10^4^ copies/mL
HbA1c	9.6 ％
β-D glucan	69.4 pg/mL
Cryptococcal antigen	negative
T-SPOT	negative
anti-Toxoplasma IgG Ab	negative
CMV antigenemia (C7-HRP)	0
HBV-DNA	negative
**Cerebrospinal fluid**	
Cell	21 /μL
Polynucleated	0 ％
Mononucleated	100 ％
Protein	77.4 mg/dL
Sugar	79 mg/dL
RPR	0.9 R.U.
ADA	2.4 U/L
Cryptococcal antigen	negative
HSV-DNA	negative
multiplex PCR	VZV positive
Cytology	negative
Culture	negative

**Abbreviations:** BUN: blood urea nitrogen, AST: aspartate aminotransferase, ALT: alanine aminotransferase, CRP: C-reactive protein, HIV: human immunodeficiency virus, anti-Toxoplasma IgG Ab: anti-Toxoplasma IgG antibody, CMV: cytomegalovirus, HBV: hepatitis B virus, HSV: herpes simplex virus, VZV: varicella-zoster virus.

**Fig. 1:**
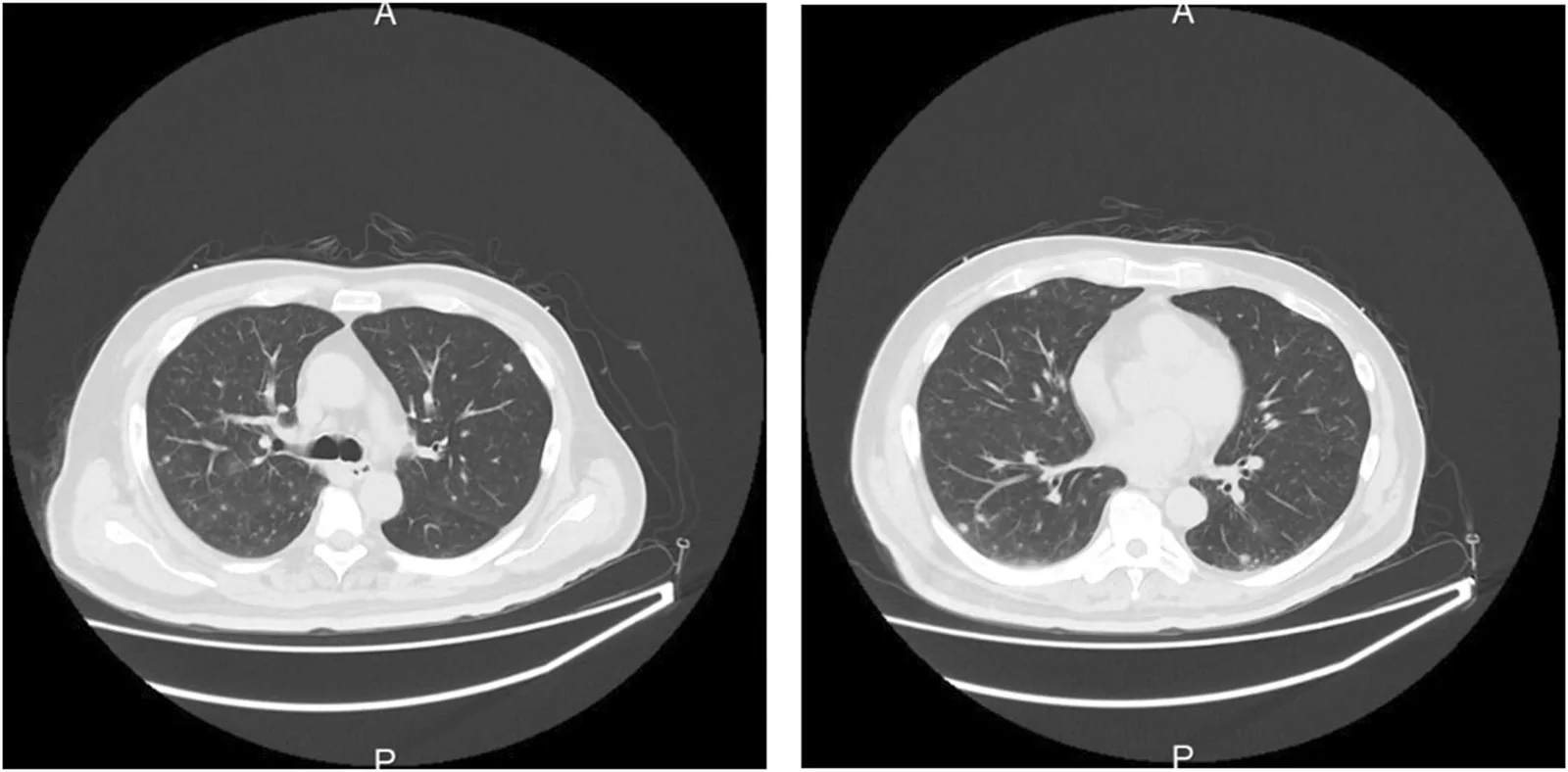
Chest computed tomography at admission revealed bilateral multiple small nodules in a hematogenous distribution.

Chest computed tomography (CT) revealed multiple, small, bilateral nodules with a hematogenous distribution (Fig. 1). The differential diagnosis included miliary tuberculosis and septic pulmonary embolism. Other opportunistic infections were also considered, including disseminated fungal infections such as histoplasmosis, cryptococcosis, coccidioidomycosis, and disseminated candidiasis; disseminated nontuberculous mycobacterial infection; viral pneumonia, including VZV; and *Pneumocystis jirovecii* pneumonia. Noninfectious etiologies, such as hematogenous pulmonary metastases, miliary-pattern lung adenocarcinoma, pulmonary diffuse large B-cell lymphoma, and pulmonary Kaposi sarcoma, were also considered.

Esophagogastroduodenoscopy demonstrated multiple, gastric erosions and ulcers, as well as esophageal candidiasis. Histopathology of a gastric biopsy specimen demonstrated the absence of typical intranuclear inclusion bodies; however, immunohistochemical staining was positive for VZV in a small number of cells. Staining for cytomegalovirus, *Treponema pallidum*, and *Helicobacter pylori* was negative. Based on these findings, DHZ with pulmonary and gastric involvement in the context of an advanced HIV infection was suspected, and intravenous acyclovir 30 mg/kg/day was begun.

**Fig. 2:**
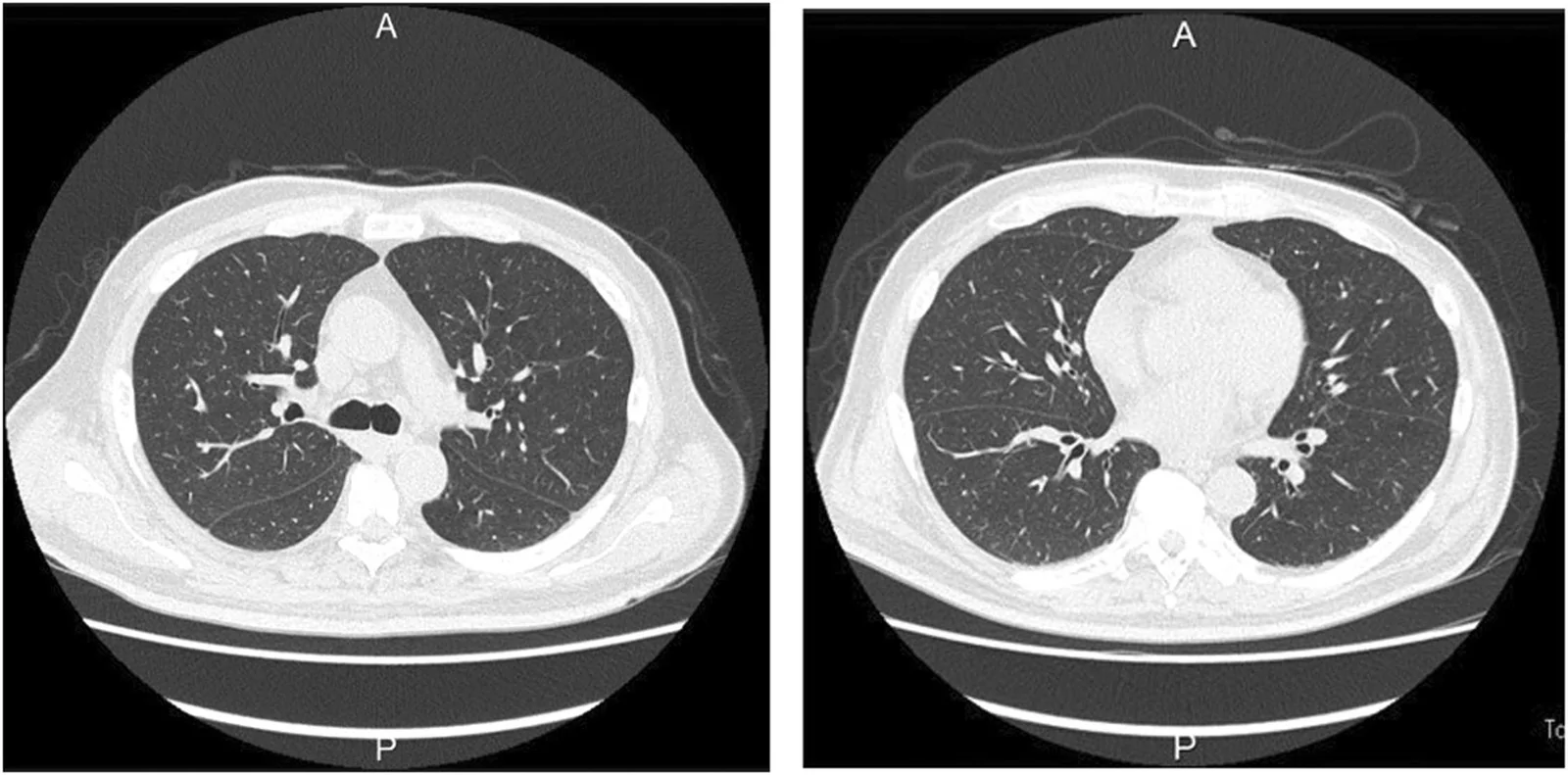
Chest computed tomography 18 days after starting intravenous acyclovir revealed that the lesions had almost disappeared.

The cutaneous lesions rapidly crusted after the antiviral therapy was begun. Remarkably, chest radiography performed several days later demonstrated regression of the bilateral, pulmonary nodules while follow-up CT performed 18 days after the start of antiviral therapy demonstrated an almost complete resolution of the lesions (Fig. 2). Therefore, pulmonary involvement by VZV was inferred from the clinical context and radiological response to antiviral therapy, rather than confirmed microbiologically. Multiple sputum acid-fast bacillus smears and cultures continued to be negative. Esophagogastroduodenoscopy performed again on day 8 of treatment demonstrated scarring at the sites of the previously observed gastric ulcers. The patient and his family did not recall any previous episode of varicella infection and denied having received a vaccination. Paired VZV serology demonstrated IgM values of 0.96 and 0.62 and IgG > 128.0 on days 5 and 22 after symptom onset.

Acyclovir was discontinued after three weeks of treatment. The esophageal candidiasis was subsequently managed with oral fluconazole. Antiretroviral therapy consisting of bictegravir, emtricitabine, and tenofovir alafenamide was begun on day 34. The patient was discharged on day 44 in good condition without any evidence of immune reconstitution inflammatory syndrome.

## Discussion

The present case illustrated several, important, clinical lessons about DHZ in immunocompromised patients with an HIV infection and a history of TNF-α inhibitor therapy.

First, pulmonary involvement in DHZ, although rare, should be considered in the differential diagnosis of diffuse, bilateral, nodular opacities, especially in patients with acquired immunodeficiency syndrome. The radiographic features of VZV pneumonia are highly variable, ranging from indistinct nodules to ground-glass opacities and reticular changes [7], [8], [9], [10]. These findings may closely resemble miliary tuberculosis, which typically presents with numerous, 2–4 mm nodules distributed throughout both lungs [11]. In our patient, the chest CT findings initially raised suspicion of miliary tuberculosis, but prompt improvement of the pulmonary lesions following antiviral therapy, combined with repeatedly negative acid-fast bacillus test results, supported the diagnosis of VZV pneumonitis rather than tuberculosis. Accurate differentiation is essential because treatment strategies and infection control measures differ substantially.

Second, gastrointestinal involvement in DHZ is extremely rare, with only sporadic cases being reported [12], [13]. Our patient presented with severe, epigastric pain four days before the appearance of the characteristic skin eruptions in line with the findings of previous reports [14]. Preceding abdominal pain may serve as an important diagnostic clue for DHZ before the appearance of characteristic skin eruptions. Endoscopy revealed multiple, gastric erosions and ulcers, and VZV immunohistochemistry confirmed viral involvement. The rapid healing of the gastric lesions following antiviral therapy lent further support to this diagnosis. Even with timely treatment, DHZ with visceral dissemination in immunocompromised individuals is associated with a mortality rate of approximately 25% [15], highlighting the importance of early diagnosis and prompt antiviral therapy.

Third, the present case underscores the complex interplay of multiple, immunosuppressive factors, particularly previous adalimumab exposure, HIV infection, and uncontrolled diabetes mellitus. While bimekizumab is not strongly associated with herpes zoster reactivation [16], multiple studies have demonstrated a significantly increased risk associated with adalimumab [4]. Given its mean terminal elimination half-life of approximately two weeks, complete drug clearance typically requires 2–4 months [17]; however, some studies of rheumatoid arthritis patients have found the drug present in blood up to six months after the discontinuation of treatment [18]. In our patient, DHZ occurred five months after he discontinued adalimumab, suggesting that residual immunosuppression may have contributed to the onset of the disease. Nevertheless, advanced HIV infection was likely a major driver of impaired cellular immunity in the present case, whereas previous adalimumab exposure may have acted as an additional contributing factor. Because uncontrolled diabetes mellitus coexisted, the relative contribution of HIV infection versus prior adalimumab exposure cannot be quantified from a single case. These findings emphasize the need to test carefully for opportunistic infections in patients with a recent history of TNF-α inhibitor therapy even if several months have lapsed since the cessation of treatment.

Finally, this case highlights the importance of comprehensive screening for latent infections, including HIV, tuberculosis, and hepatitis B, prior to initiating biologic therapy. Our patient had a history of syphilis and hepatitis B virus infection but had not been screened for HIV before starting adalimumab therapy. This omission may have delayed recognition of his immunocompromised state.

In conclusion, DHZ should be considered in the differential diagnosis of diffuse, bilateral, pulmonary nodules in immunocompromised patients, particularly those with an HIV infection and a history of TNF-α inhibitor therapy. Careful, radiological follow-up after the start of antiviral therapy in parallel with acid-fast bacillus testing helps differentiate the condition from miliary tuberculosis. Clinicians should remain vigilant for atypical manifestations of infectious diseases in patients who are receiving or have recently discontinued biologic therapy.

## Ethics declaration

Written informed consent to take part in the study and to publish the article has been obtained from all participants or their legal representatives. The privacy rights of participants have been observed.

This study was performed in compliance with relevant laws, regulatory frameworks and guidelines where the research took place. Ethics committee approval was not required under relevant laws and institutional guidelines. Ethics committee approval was not required for this single-patient case report in accordance with the institutional guidelines of Tokyo Metropolitan Cancer and Infectious Diseases Center Komagome Hospital.

This research follows the CARE guidelines and the CARE checklist.

## CRediT authorship contribution statement

**Shuhei Tada:** Conceptualization, Writing – original draft. **Kazuaki Fukushima:** Supervision, Writing – review & editing. **Yota Aizawa:** Writing – review & editing. **Shota Kodaira:** Writing – review & editing. **Seowoong Jung:** Writing – review & editing. **Masaru Tanaka:** Writing – review & editing. **Taiichiro Kobayashi:** Writing – review & editing. **Ryoko Sekiya:** Writing – review & editing. **Atsushi Ajisawa:** Supervision, Writing – review & editing. **Akifumi Imamura:** Supervision, Writing – review & editing.

## Funding

This research did not receive any specific grant from any funding agency in the public, commercial or not-for-profit sector.

## Declaration of Competing Interest

The authors declare that they have no known competing financial interests or personal relationships that could have appeared to influence the work reported in this paper.

## Data Availability

The data used to support the findings of this study are available from the corresponding author on reasonable request.
